# Correlation of Parasite Burden, kDNA Integration, Autoreactive Antibodies, and Cytokine Pattern in the Pathophysiology of Chagas Disease

**DOI:** 10.3389/fmicb.2019.01856

**Published:** 2019-08-21

**Authors:** Moisés Wesley, Aline Moraes, Ana de Cássia Rosa, Juliana Lott Carvalho, Tatiana Shiroma, Tamires Vital, Nayra Dias, Bruna de Carvalho, Doralina do Amaral Rabello, Tatiana Karla dos Santos Borges, Bruno Dallago, Nadjar Nitz, Luciana Hagström, Mariana Hecht

**Affiliations:** ^1^Interdisciplinary Laboratory of Biosciences, Department of Pathology, Faculty of Medicine, University of Brasília, Brasília, Brazil; ^2^Genomic Sciences and Biotechnology Program, Catholic University of Brasília, Brasília, Brazil; ^3^Department of Pathology, Faculty of Medicine, University of Brasília, Brasília, Brazil; ^4^Laboratory of Molecular Pathology of Cancer, Department of Pathology, Faculty of Medicine, University of Brasília, Brasília, Brazil; ^5^Laboratory of Cellular and Molecular Immunology, Department of Pathology, Faculty of Medicine, University of Brasília, Brasília, Brazil; ^6^Laboratory of Animal Welfare, Faculty of Agronomy and Veterinary Medicine, University of Brasília, Brasília, Brazil

**Keywords:** Chagas disease, pathophysiology, correlation analysis, parasite burden, autoimmunity

## Abstract

Chagas disease (CD), caused by the protozoan *Trypanosoma cruzi* (*T. cruzi*), is the main parasitic disease in the Western Hemisphere. Unfortunately, its physiopathology is not completely understood, and cardiomegaly development is hard to predict. Trying to explain tissue lesion and the fact that only a percentage of the infected individuals develops clinical manifestations, a variety of mechanisms have been suggested as the provokers of CD, such as parasite persistence and autoimmune responses. However, holistic analysis of how parasite and host-related elements may connect to each other and influence clinical outcome is still scarce in the literature. Here, we investigated murine models of CD caused by three different pathogen strains: Colombian, CL Brener and Y strains, and employed parasitological and immunological tests to determine parasite load, antibody reactivity, and cytokine production during the acute and chronic phases of the disease. Also, we developed a quantitative PCR (qPCR) protocol to quantify *T. cruzi* kDNA minicircle integration into the mammalian host genome. Finally, we used a correlation analysis to interconnect parasite- and host-related factors over time. Higher parasite load in the heart and in the intestine was significantly associated with IgG raised against host cardiac proteins. Also, increased heart and bone marrow parasitism was associated with a more intense leukocyte infiltration. kDNA integration rates correlated to the levels of IgG antibodies reactive to host cardiac proteins and interferon production, both influencing tissue inflammation. In conclusion, our results shed light into how inflammatory process associates with parasite load, kDNA transfer to the host, autoreactive autoantibody production and cytokine profile. Altogether, our data support the proposal of an updated integrative theory regarding CD pathophysiology.

## Introduction

Chagas disease (CD) is an endemic disease in South and Central America, caused by the protozoan *Trypanosoma cruzi*. It is considered a neglected tropical endemic by WHO, yet it threatens 25 million people worldwide and causes significant morbidity and mortality with more than 10,000 deaths per year (World Health Organization, 2018). Importantly, around 10% of CD burden currently affects non-endemic regions, such as developed countries, due to recent disease spread beyond its original vector-associated boundaries. Therefore, CD has become a global issue (World Health Organization, 2018).

Acute CD usually runs asymptomatically, with some individuals exhibiting nonspecific and mild symptoms, despite elevated parasite load (Teixeira et al., 2011a; Costa et al., 2017). In the absence of treatment, the 4–8 week acute phase usually resolves spontaneously. Nevertheless, infection persists with low parasitemia and the disease progresses to an unpredictable chronic phase, which begins with no symptoms but can evolve to cardiac, digestive, or mixed (both cardiac and digestive) forms in about 30–40% of the patients, requiring complex treatment (Gironès et al., 2005; Costa et al., 2017; Zrein et al., 2018).

In this respect, the discrepancies between parasite load and the emergence of clinical manifestations underscore the intricate aspect of CD pathogenesis, requiring further investigation. The hypothesis of parasite persistence attributes a direct role of the parasite in the maintenance of chronic inflammation and consequent tissue damage observed in individuals infected with *T. cruzi* (Cruz et al., 2016). In this sense, there is little controversy that host cell lysis caused by *T. cruzi* has a role in the cardiac damage observed in some individuals (Bonney and Engman, 2015). Autoimmunity is also pointed as a relevant phenomenon in CD pathogenesis and there is growing consensus that autoreactivity is triggered by *T. cruzi* infection.

A variety of mechanisms have been suggested as the means by which the parasite can provoke autoimmune response, such as molecular mimicry, bystander activation (De Bona et al., 2018), and the transfer to the host genome of the *T. cruzi* kinetoplast DNA (kDNA) (Teixeira et al., 2011b), a DNA found in a specialized portion of the mitochondrion. Currently, the kDNA integration hypothesis is questioned by some researchers, due to the unilateral retraction of the work of Nitz et al. (2004). However, previous experimental evidence pointing toward the integration of parasite kDNA into vertebrate genomes had been acquired using different methodologies, such as fluorescent *in situ* hybridization (FISH) and genomic Southern blotting (Teixeira et al., 1991, 1994; Simões-Barbosa et al., 1999, 2006). Furthermore, the original work of Nitz et al. (2004) was subsequently supported by additional studies involving humans, rabbits, and chickens (Hecht et al., 2010; Teixeira et al., 2011a, 2012; Guimaro et al., 2014), suggesting that such event is not restricted to a given host species or parasite strain. To date, no experimental data refuting the original observations of kDNA integration have been published. Finally, how the integration event influences clinical manifestations of CD is still largely unknown.

In spite of all the acquired knowledge regarding different elements of CD pathophysiology, comprehensive analysis of how different disease aspects relate to each other and influence the clinical outcome is still scarce in the literature. Given the controversy regarding the major mechanisms related to CD pathogenesis and how they correlate with each other, this study aimed to shed light into CD pathobiology, investigating whether parasite-related (parasite load and parasite strain), as well as host-related factors (gender, immune response, autoantibody production, and kDNA integration) contribute to disease progression and clinical manifestations. Our correlation analysis sheds important light into how autoimmunity (autoreactive antibody production) associates with parasite load, kDNA transfer to the host and interferon levels, and supports an updated integrative theory regarding CD pathophysiology.

## Materials and Methods

### *Trypanosoma cruzi* and Macrophage Culture

Trypomastigote forms of *T. cruzi* Colombian, CL Brener and Y strains were grown in murine cardiac L6 cell line, cultured with Dulbecco’s Modified Eagle Medium (DMEM), supplemented with 10% v.v. Fetal Bovine Serum (FBS), 100 IU/ml penicillin, and 100 μg/ml streptomycin in a humidified atmosphere with 5% CO_2_ at 37°C. Amastigote forms were obtained from trypomastigote culture after centrifuging cell culture supernatant containing trypomastigotes at 5,000 rpm for 15 min. Parasite pellet was resuspended in DMEM, pH 5.0 and incubated for 5 h in a humidified atmosphere with 5% CO_2_ at 37°C. Then, parasite culture was centrifuged again at 5,000 rpm for 15 min and resuspended in complete DMEM medium, pH 7.4 supplemented with 5% v.v. FBS and incubated overnight in a humidified atmosphere with 5% CO_2_ at 37°C.

Murine macrophage cell line J774A.1 (ATCC number: TIB-67), obtained from the European collection of cell cultures (ECACC) was grown as previously described (Simões-Barbosa et al., 2006). J774A.1 cells were infected with 1 × 10^6^ trypomastigotes, at a ratio (*T. cruzi*:J774A.1) of 5:1.

### Experimental Groups and Infection

BALB/c mice were obtained from the animal facility of the University of Brasilia Medical School. Animals were kept at a controlled temperature under a 12/12 h light/dark cycle with free access to food and water. Mice were infected with 1 × 10^4^ intraperitoneal (i.p.) injection of trypomastigote forms of *T. cruzi* from Colombian (DTU I), Y (DTU II) or CL Brener (DTU VI) strains. At day 7 post infection, blood parasitemia was assessed in tail blood by direct parasite observation under Olympus BX51 microscope, model U-LH100HG (Olympus^®^), with a 40X objective. If no parasitemia was detected, the procedure was repeated daily until confirmation of the presence of the parasite.

Animals were euthanized at 30- or 100-days post-infection (dpi), corresponding to the acute and chronic phase of the disease (Supplementary Table S1). All experimental protocols were performed in accordance with the guidelines for the human use of laboratory animals established at our Institution. Animal work was approved by the University of Brasília Animal Research Ethics Committee (CEUA), under protocol 150406/2015.

### Serum and DNA Sample Obtention

At the established endpoints (Supplementary Table S1), mice were sacrificed and about 200 μl of the collected blood by cardiac puncture was used to obtain the serum. Cardiac, intestinal, and medullary tissue were also collected. Cardiac and intestinal tissue were used for DNA isolation and processed for histology. Medullary tissue was used for DNA isolation only.

### Total Genomic DNA Isolation and Quantitative PCR (qPCR) Analysis

To determine the parasitic load and the accumulation of kinetoplast DNA (kDNA) minicircle integration into the host genome, total DNA extraction was performed with the Mini Spin Plus extraction kit (Biopur^®^), according to the manufacturer’s recommendations. Total DNA quality and integrity were verified by conventional PCR using the constitutive β-actin gene as a target.

qPCR was used to quantify *T. cruzi* nuclear (nDNA) or mitochondrial DNA (kDNA) in test samples. Standard curves were constructed from different *T. cruzi* total DNA concentrations using serial dilutions of parasite total DNA from 10^5^ to 10^−2^ parasite equivalents. Standard curve efficiencies were similar for both pairs of primers. nDNA detection primers showed 96.4% efficiency, and kDNA detection primers showed 96.6% efficiency. Blank (DNA-free water), negative, and positive controls (DNA from non-infected and infected samples) were included in all plates and used to validate the results. To avoid differences in multi-plate measurements, two *T. cruzi* total DNA samples (corresponding to 10^4^ and 10^1^ parasite equivalents) were included in each plate and used as calibrators, applying a between-run correction factor, according to Ruijter et al. (2015).

We amplified nDNA with TCZ3 (5′ TGC ACT CGG CTG ATC GTT T3′) and TCZ4 (5′ ATT CCT CCA AGC AGC GGA TA3′) primers, which have high sensitivity and specificity for all *T. cruzi* lineages (Ndao et al., 2000). The following conditions were used: 50°C for 2 min, 95°C for 10 min, and 40 cycles of 95°C for 15 s, 57°C for 60s, and 72°C for 10 s. kDNA was amplified using S36 (5′GGT TCG ATT GGG GTT GGT G3′) and S67rev (5′GAA CCC CCC TCC CAA AAC C3′) primers, which target the most frequent sequences found in kDNA-host DNA chimeras deposited in databases (Hecht et al., 2010; Teixeira et al., 2011a,b). qPCR conditions were as follows: 50°C for 2 min, 95°C for 10 min, and 40 cycles of 95°C for 15 s, 60°C for 45 s, and 72°C for 10 s. All qPCR experiments were carried out using 200 ng of total DNA, 0.2 μM of each primer, and 10 μl Power SYBR^®^ Green PCR Master Mix (Applied Biosystems, CA, USA) in a final volume of 20 μl (adjusted with DNAse and RNAse-free water). The reactions were carried out in 96-well plates (Optical 96-Well Reaction Plate, MicroAmp^®^), in technical and biological triplicates, in the 7,500 Real-Time PCR System thermal cycler (Applied Biosystems, CA, USA).

### Analysis of kDNA Integration

To verify the accumulation of parasite kDNA integration in the samples, the ratio of kDNA/nDNA of each *T. cruzi* strain used in this study (five *pools* of 1.10^7^ parasites for each strain) was determined and used as threshold. Superior kDNA/nDNA ratios were considered indicators of parasite genomic integration. To further validate our protocol, we then assessed the transfer of *T. cruzi* kDNA minicircles into the host genome using J774A.1 mouse macrophages at 7, 15, and 30 days post-infection (dpi). After infection, cells were treated or not with the trypanocide benznidazole (322 μM, once a week, for 4 weeks). Parasite elimination was confirmed visually and by qPCR. Amplification data were compared to genomic *Southern* blot, performed as follows: 20 μg of the DNA samples were added to a mix containing 10 μl of 10X enzyme buffer, 4 μl of NSiI enzyme, 4 μl of BSA (Bovine Serum Albumin), in a final volume of 100 μl with ultrapure water. A total of 100 ng of the parasite DNA was used. Of interest, the NSiI enzyme performs a cut in each of four conserved regions of the kDNA minicircle, resulting in a band of 0.36 kb. Thus, larger or smaller bands than this size correspond to kDNA integration (Simões-Barbosa et al., 2006).

After electrophoretic separation in 0.8% agarose gel, the digested DNA was transferred to a positively charged nylon membrane (PALL Biodyne BD) by the capillary transfer method (Sambrook et al., 1989). Membranes were labeled with probes prepared with Biotin 3′End DNA Labeling Kit (ThermoFisher, MA, USA), according to manufacturer’s recommendations. The probes corresponded to a mix of three primers specific to kDNA minicircles: S36, S35r, and S67 (Hecht et al., 2010). The probes attached to the specific sequence were detected by Immobilized Nucleic Acid Detection Chemiluminescent kit (ThermoFisher, MA, USA), according to manufacturer’s instructions. After the addition of bioluminescent reagents, the membrane was exposed to X-ray film (KODAK T-MAT) for 30 min. The film was revealed in a dark room.

### Indirect Enzyme-Linked Immunosorbent Assay (ELISA)

Production of parasite-reactive or autoreactive antibodies was assessed by ELISA using serum samples. Immunoglobulin M (IgM) was measured during the acute phase, while immunoglobulin G (IgG) was measured during the chronic phase. Briefly, plates were sensitized with 50 μl per well of the parasite, cardiac or intestinal antigens solubilized in 1X PBS buffer pH 7.4 and incubated overnight in a humidified atmosphere at 37°C. Then, 150 μl of 1X Milk-PBS (pH 7.4, 5% w.v. skim milk) was added. Serum samples were diluted 1:100 using 1X Milk-PBS (pH 7.4, 2% w.v. skim milk) and added to the wells. After 2 h incubation at 37°C in a humid chamber, plates were washed and 50 μl/well of the secondary antibody was added. Anti-mouse IgG (Sigma-Aldrich) conjugated to alkaline phosphatase was used at 1:2,000 dilution, and anti-mouse IgM conjugated to alkaline phosphatase (Sigma-Aldrich) was used at 1:1,000 dilution. Each well then received 50 μl/well of revealing solution (pnPP-p-nitrophenol phosphate – diluted in diethanolamine buffer pH 9.8) and, after 12-min incubation in the dark, reading was performed at 405 nm using BioTeK^®^-Synergy HT spectrophotometer. Cut-off values were determined for each antigen using the (MEAN + 3 × SD) of negative controls, where SD is the standard deviation (Supplementary Figure S1; Lardeux et al., 2016). Samples with superior optical density (OD) (>10% above cut-off) were considered positive. Likewise, samples with inferior OD compared to cut-off value were considered negative.

### Cytokines Measurement by Flow Cytometry

Interleukin-2 (IL-2), interleukin-4 (IL-4), interleukin-5 (IL-5), tumor necrosis factor (TNF), and interferon-γ (IFN-γ) were measured with the Cytometric Bead Array (CBA) Mouse Th1/Th2 Cytokine Kit (BD), following the manufacturer’s instructions. LSRFortessa™ BD cytometer was used for data acquisition, and FCAP 3.0 software (BD Biosciences^®^, USA) was used for data analysis.

### Histological Analysis

Amastigote nest detection, leukocyte infiltration, and tissue lesion were performed according to Ribeiro et al. (2016). After sample collection, fixation, and paraffin embedding, three 5 μm sections of different parts of each tissue were obtained and stained with Hematoxylin and Eosin (HE). The slides were analyzed under an Olympus BX51 U-LH100HG optical microscope. ScanScope^®^ (Aperio) was used to record the images. Tissue alterations were classified according to Castro-Sesquen et al. (2011) criteria, with minor modifications. Parasitism analysis considered the total number of amastigote nests observed in each slide, being: 0 = absent, 1 = basal (1–5 amastigotes nests), 2 = moderate (6–10 nests), 3 = severe (>10 nests). The intensity of the inflammatory process was classified as: 0, normal; 1, mild myocarditis (lymphocytes seen in 2–15% of the entire section); 3, moderate (lymphocytes seen in 20–60% of the section); and 4, severe myocarditis (lymphocytes seen in >70% of the section). After that, the mean of inflammatory infiltrates observed in 15 fields of each slide was calculated, generating a final criterion: 0–0.3 = normal; 0.4–1.0 = mild; 1.1–2 = moderate; 2.1–3 = severe.

### Statistical Analysis

The experimental design was completely randomized with a factorial scheme comprising 16 treatments related to the combinations of the following variables: stage (acute or chronic), strain (uninfected, Colombian, CL Brener or Y), and sex (male or female). Five replicates were used per treatment. Variables were assessed for normality using the Shapiro-Wilk test. Non-normal distribution variables were transformed (log or root transformation) or analyzed by the PROC GLIMMIX procedure. Variables with normal distribution were submitted to analysis of variance (PROC GLM) followed by comparison of means by Tukey test at 5% of significance. Qualitative dependent variables were submitted to the Chi-square test. Pearson analysis (PROC CORR) was performed with the quantitative data to ascertain the relationships among the results of the measured variables and the figure was built using the R core package (Wei et al., 2017; R Core Team, 2019). The principal component analysis was carried out with total raw data using PROC CORR procedure. All statistical analyses were performed using the SAS^®^ program (version 9.3, Cary, North Carolina) at 5% significance level. Data are presented as means and standard deviation, or absolute values.

## Results

### Parasite Load Quantification

**Figure 1:**
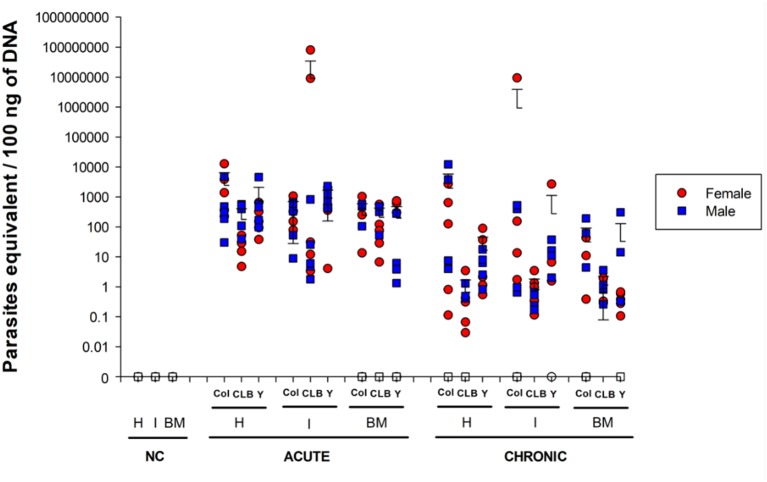
Parasite burden in mice infected with different *Trypanosoma cruzi* DTU lineages. Heart (H), intestine (I), and bone marrow (BM) DNA samples were obtained from mice infected with Colombian (Col), CL Brener (CLB) or Y strains at 30 and 100 days post infection (acute and chronic phases, respectively). DNAs were used as template in qPCR reactions using specific primers for *T. cruzi* nuclear DNA. Symbols indicate absolute levels, and bars indicate mean + standard deviation. Solid symbols indicate animals with positive parasite detection. No statistical difference was found by ANOVA for any of the evaluated parameters. Statistically significant differences to non-infected, negative control (NC) samples were not presented.

Successful infection was observed in all animals, regardless of parasite strain, as revealed by cardiac and intestinal qPCR analysis during the acute phase (Figure 1). However, parasite detection in the bone marrow was negative in 40% of the mice infected with the Colombian strain, 10% of the animals infected with CL Brener, and 30% of the animals infected with Y strain, revealing a more heterogeneous parasite infection of bone marrow (Supplementary Figure S2).

During the chronic phase of the infection, parasite distribution showed greater variation among the different sites according to the infecting strain. Namely, 90% of mice infected with *T. cruzi* Colombian strain had positive parasite detection in the heart and intestine, but only 60% of the infected mice had positive parasite detection in the bone marrow. All CL Brener-infected mice had positive parasite detection in the intestine and bone marrow, but only 60% of heart samples of these animals tested positive for *T. cruzi*. All animals infected with Y strain showed positive parasite genomic amplification of cardiac tissue, whereas 90% of the animals had positive parasite detection in the bone marrow and 70% in the intestinal tissue. Despite the observed differences in the percentage of animals with positive qPCR parasite genome amplification, we only verified statistically significant difference when comparing the bone marrow samples of animals infected with the Colombian *versus* CL Brener strains (*p* = 0.008), and when comparing the intestine samples of animals infected with the Y *versus* Colombian and CL Brener strains (*p* < 0.05). Interestingly, the host sex did not significantly alter parasite detection pattern at the analyzed sites (Supplementary Table S2).

qPCR data were also used to quantitatively analyze parasite load in each tissue (Figure 1). A large discrepancy of values obtained for some animals of the same experimental group was detected, resulting in the absence of statistical significance between the acute and the chronic phase, regardless of the *T. cruzi* strain. Nevertheless, an overall reduction in the number of parasites was observed in chronically infected animals, which is consistent with previous observations (Pack et al., 2018; Mateus et al., 2019). Also of note, animals infected with the CL Brener strain presented relatively low parasite loads in all analyzed tissues during the chronic phase (heart: 0.66 ± 1.05 equivalent parasites/100 ng total DNA, intestine: 0.80 ± 10.02 equivalent parasites/100 ng total DNA, bone marrow: 1.15 ± 1.08 equivalent parasites/100 ng of total DNA).

### kDNA Integration Analysis

Initially, we determined the ratio of kDNA minicircles to nDNA in *T. cruzi* amastigotes, using qPCR to quantify DNA contents in pools of the three *T. cruzi* strains investigated (Supplementary Figure S3A). Then, we established a threshold based on the kDNA/nDNA ratio obtained for each strain, considering the maximum standard deviation. Superior kDNA/nDNA ratios indicated parasite integration into the host genome.

Before analyzing animal samples, we further confirmed our quantitative kDNA integration method using J774A.1 mouse macrophages at 7, 15, and 30 dpi. Interestingly, during the observed period, we detected a gradual increase in the kDNA/nDNA ratio, which was below the threshold at day 7, similar to the threshold at day 15 and superior to the threshold at day 30. Those observations were confirmed using *Southern* blot (Supplementary Figures S3B,C), where variations in band pattern were detected as kDNA insertions occurred. We also assessed *T. cruzi* kDNA integration after submitting cell cultures to benznidazole treatment, which efficiently eliminated the parasite, as confirmed by nDNA qPCR. Strikingly, while *T. cruzi* nDNA was not detected at day 30 post infection/treatment, kDNA was still detected in treated cell cultures, according to both qPCR and *Southern* blot data.

After validating our kDNA integration analysis protocol *in vitro*, we started to investigate kDNA integration in our *in vivo* experimental groups. We observed a higher percentage of kDNA integration in the cardiac tissue of acute phase samples compared to chronic phase counterparts (*p* = 0.0003) (Table 1). Furthermore, we detected a strain-specific variation in kDNA integration capacity, as follows: in acute phase samples, 80% of the Colombian strain-infected mice showed positive kDNA integration in cardiac tissue, compared to 100% of the CL Brener strain-infected group and 40% of Y strain-infected animals. In the chronic phase, kDNA integration was less frequent in the cardiac tissue of CL Brener-infected group, in which no kDNA integration was detected. kDNA integration in different tissues varied according to the parasite strain. Overall, it was noted that there is a higher possibility of the event occurring in the heart and bone marrow (*p* = 0.007 and *p* = 0.003, respectively) of animals during chronic infection with the Colombian strain compared to the other groups. More specifically, heart samples from Y-infected mice presented significantly lower percentage of positive kDNA detection, compared to CL Brener heart samples, during the acute phase (*p* < 0.05). During the chronic phase, both Y- and CL Brener-infected cardiac samples presented a significantly lower percentage of positive kDNA integration, compared to Colombian-infected heart samples (*p* < 0.05). Finally, bone marrow samples from CL B- and Y-infected mice presented significantly lower percentage of positive kDNA detection, compared to Col-infected group (*p* < 0.05) (Supplementary Figure S4).

**Table 1:** Percentage of animals with positive kDNA integration.

Tissue sample	Acute	Chronic	*p*
Heart	73.3% a	30.0% b	0.003
Intestine	43.3% a	36.6% a	ns
Bone marrow	40.0% a	36.6% a	ns

*ns: non significant. According to the chi-square test, experimental groups with a statistically similar percentage of kDNA integration were designated with the same letter (a or b)*.

**Figure 2:**
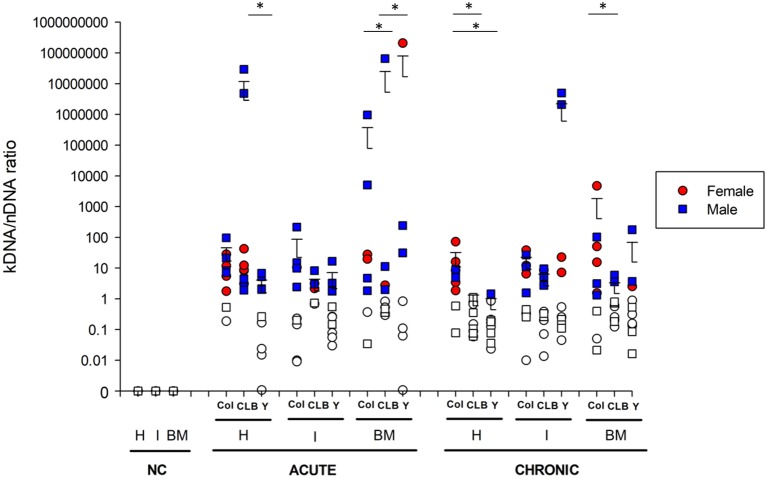
Integration of *Trypanosoma cruzi* kDNA minicircles in different tissues of infected mice. kDNA minicircle integration was assessed in different anatomical sites of animals infected with Colombian (Col), CL Brener (CLB) or Y strain. Cardiac (H), intestinal (I), and medullar (BM) samples of infected animals were collected during the acute and chronic phases of the infection. Non-infected, negative controls (NC) were also analyzed. Solid symbols indicate animals with positive kDNA integration. The cut-off was established based on kDNA/nDNA ratio from amastigote *pools* of each *T. cruzi* strain. Values greater than the threshold were considered indicators of parasite genomic integration. Symbols indicate absolute levels, and bars indicate mean + standard deviation. The asterisks represent statistical difference (*p* < 0.05) among the strains found by ANOVA for each tissue, in each infection phase. Statistically significant differences to the non-infected, negative control (NC) samples were not presented.

Surprisingly, it was observed that the integration of kDNA in the intestine occurs preferentially in male mice when compared to females (*p* = 0.04) (Supplementary Table S3). Furthermore, Y strain presented no kDNA integration in the cardiac and intestinal tissues of female mice during the acute phase, different from other parasite strains (Figure 2). During the chronic phase, kDNA integration was still negative for female intestine samples, but one female tested positive for kDNA integration in cardiac samples of Y strain infection. Male and female mice presented low, but similar positivity for Y strain kDNA integration during the acute and chronic infection. Finally, when analyzing the total percentage of kDNA integration detected in each tissue, regardless of the parasite strain, we verified that the average percentage of animals with positive integration during the chronic phase is similar among the evaluated tissues (Table 1).

Further insights into kDNA integration were obtained from quantitative analysis of *T. cruzi* kDNA minicircle integration (Figure 2). Interestingly, quantitative evaluation of the kDNA integration events indicated that host sex does not interfere in the number of kDNA integrations, neither in the acute or chronic phase of infection. Nevertheless, significant differences were detected when comparing the number of kDNA integrations of different tissues according to the infecting strain, both at acute and chronic phases of CD. Even though great intragroup variability was detected, some observations can be highlighted: (1) at 30 dpi, animals infected with the CL Brener strain showed a higher amount of kDNA integration in heart than those infected with the Y strain (*p* < 0.05); (2) also, CL Brener strain showed less frequent kDNA integrations in the host bone marrow at 30 dpi (*p* < 0.001) compared to Colombian and Y strains; (3) except for Colombian strain, a significant reduction in the number of integrations in the heart of all animals was detected (*p* < 0.001) over time. During the chronic phase, statistically significant (*p* < 0.05) differences were also observed, as follows: (1) Colombian strain showed greater number of kDNA integrations in heart samples, compared to the other strains; (2) in the bone marrow, Colombian strain showed higher amount of kDNA integrations, compared to CL Brener samples.

### Immune Response Analysis

**Figure 3:**
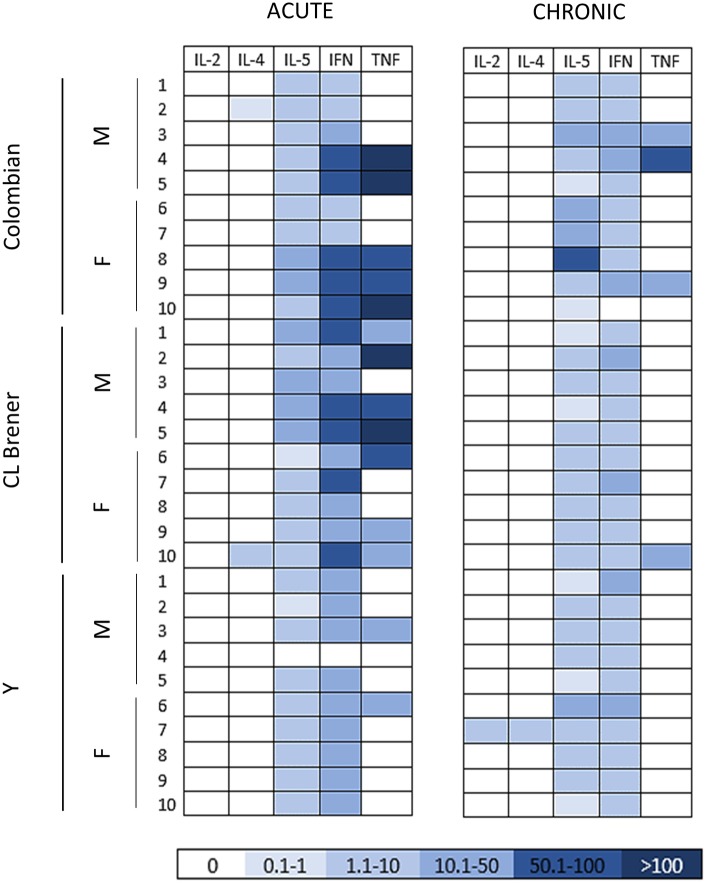
Cytokine profile in mice infected with different *Trypanosoma cruzi* strains during the acute and chronic phases of Chagas disease. The heat map shows the concentration of the cytokines (pg/ml) detected in the serum of infected mice at 30 and 100 dpi. Note a decline in serum cytokine concentration, markedly in pro-inflammatory cytokines, as the infection progressed from the acute to the chronic phase. M: male samples. F: female samples. IL: interleukin. INF: interferon γ. TNF: tumor necrosis factor.

To characterize host immune response against *T. cruzi*, first we assessed cytokine production in serum (Figure 3). Namely, TNF, IFN-γ, IL-5, IL-4, and IL-2 were quantified using flow cytometric analysis. In acute CD, a pro inflammatory cytokine production pattern was detected, with superior production of TNF and IFN-γ in mice infected with Colombian (TNF 64.5 ± 78.9 pg./ml, IFN 36.9 ± 3.0 pg./ml) and CL Brener (TNF 45.5 ± 50.4 pg./ml, IFN 47.5 ± 13.4 pg./ml) strains (Supplementary Table S4). As the infection progressed to the chronic phase, a marked decline in serum cytokine concentration was observed, even though the infection is persistent. During this period, Colombian strain-infected mice presented a sex-related variation: while IL-5 production was higher in females than in male samples (*p* < 0.05), TNF had a superior concentration in males compared with female samples (*p* < 0.05) of the same experimental group.

**Figure 4:**
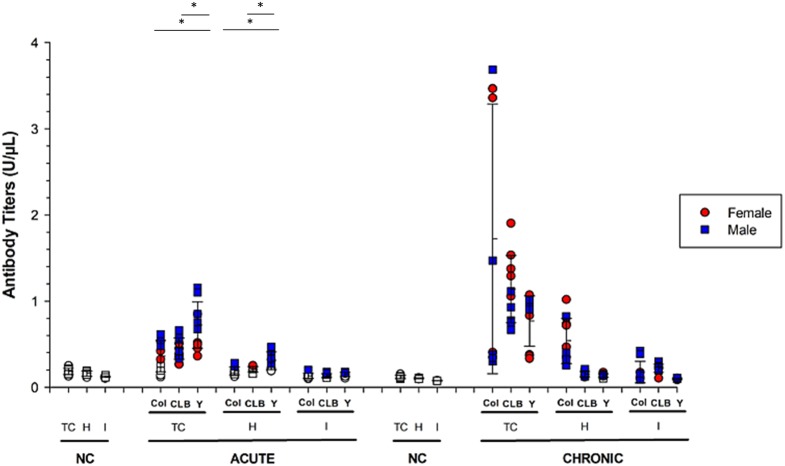
Humoral response raised against parasite- and host-derived antigens in mice infected by different strains of *T. cruzi*. Serum immunoglobulins reactivity against parasite (TC), cardiac (H), and intestinal (I) antigens was assessed using ELISA during the acute (IgM) and chronic (IgG) phases Chagas disease. The graphs show animals infected with Colombian (C), CL Brener (CL) or Y strains. Solid symbols indicate animals with positive serology. Values greater than the threshold established for each antigen were considered positive and are presented as solid symbols. Data are presented as absolute levels, and bars indicate mean + standard deviation. The asterisks represent statistical difference (*p* < 0.05) found by ANOVA. Statistically significant differences to negative control (non-infected animals; NC) samples were not presented.

Then, the humoral response was assessed by the detection of specific antibodies against *T. cruzi*, as well as cardiac and intestinal autoantigens (Figure 4). The production of IgM immunoglobulins against parasite antigens revealed no significant difference between male and female mice, regardless of the infecting parasite strain. In mice infected with the Colombian strain, anti-*T. cruzi* IgM was detected in 60% of the animals, which significantly differed (*p* = 0.04) from anti-*T. cruzi* IgM production of animals infected with the other strains (90% for CL Brener and 100% for Y strain) during the acute phase (Supplementary Figure S5). Still considering CD acute phase, it was possible to verify that 60% of the mice infected with the Y strain produced antibodies against cardiac and intestinal antigens, whereas such autoimmune recognition was much smaller in any other group (*p* < 0.05). In this respect, it was observed that all males infected with the Y strain produced antibodies against both antigens, while only one female was reactive (*p* < 0.05).

In the chronic phase of the infection (100 dpi), all animals produced IgG antibodies against *T. cruzi* and intestinal proteins. In addition, Y strain-infected mice presented significantly lower percentage of animals (60%) showing positive reaction against heart antigens, compared to Colombian- (100%) and CL Brener-infected mice (90%) (*p* < 0.05).

When performing quantitative analysis of antibody production against parasite- or host-derived antigens (Figure 4), it was found that – during CD acute phase – animals infected with the Y strain produced higher amounts of anti-*T. cruzi* and anti-cardiac protein IgM, compared to animals infected with the other strains (*p* < 0.05). Interestingly, the highest antibody rates detected in the Y strain-infected mice were obtained from male mice and reacted with both *T. cruzi* (*p* = 0.01) and cardiac antigens (*p* = 0.007), compared to female counterparts.

In chronic CD, male mice infected with the Y strain kept producing high rates of anti-*T. cruzi* immunoglobulins compared to females (*p* = 0.03). The opposite pattern was observed for CL Brener strain, in which infected females produced higher amounts of reactive antibodies against *T. cruzi* antigens (*p* = 0.01). The production of IgG against cardiac tissue was higher in animals infected with the Colombian strain, without statistical difference according to sex (*p* > 0.05).

Considering the production of antibodies against host-derived intestinal proteins, low autoreactivity in a few animals was detected in the acute phase, but all animals started producing autoreactive IgG during chronic CD. Similar antibody production levels were detected among the different experimental groups (*p* > 0.05), regardless of host gender.

### Histological Analysis

**Figure 5:**
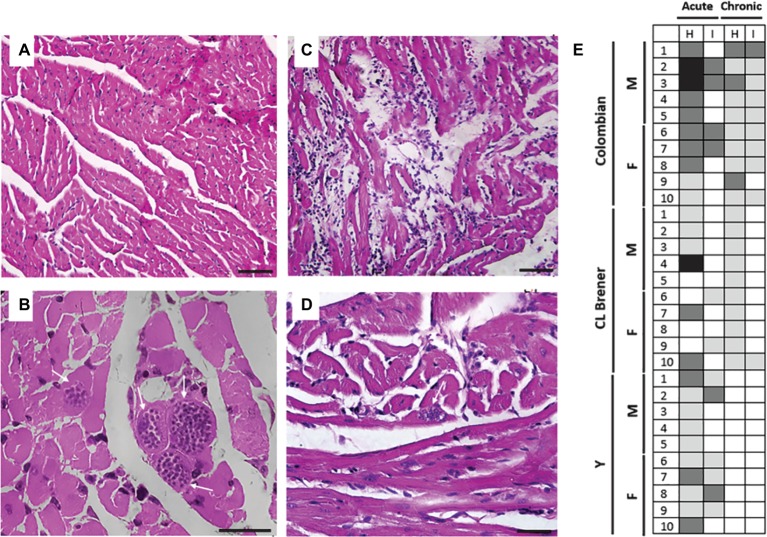
Tissue parasitism and inflammatory process in mice infected with different Trypanosoma cruzi strains. Compared to healthy animals, which presented normal cardiac tissue **(A)**, animals infected with Colombian T. cruzi strain presented amastigote nests (white arrows) in the heart during CD acute phase **(B)**. During CD chronic phase, inflammatory infiltrate and lysis of cardiac fibers could be observed **(C)**, as well as less frequent parasite nests (white arrow) **(D)**. **(E)** The intensity of leukocyte infiltration was analyzed in cardiac (H) and intestine tissue (I) and considered as follows: 0–0.2 normal (white), 0.3–1.0 mild (light gray), 1.1–2.0 moderate (dark gray), 2.1–3.0 severe (black). M: male samples. F: female samples. Bars: 10 μm.

To further understand the complex relationship between *T. cruzi* tissue parasitism, autoimmunity, cytokine production, and tissue damage, we assessed the presence of amastigote nests, as well as the intensity of inflammatory infiltration and tissue reaction (hemorrhage and necrosis) in cardiac and intestinal tissues of infected animals (Figures 5A–D). Despite qPCR analysis, which indicated positive *T. cruzi* parasitism in cardiac and intestinal tissues, intracellular parasite nests were only visually detected in the cardiac tissue of 80% of animals infected with the Colombian strain during the acute phase and 40% of the animals infected with such strain during the chronic phase. No amastigote nests were seen in the gut nor in the cardiac tissues of animals infected with CL Brener and Y strains.

The inflammatory process (Figure 5E) was more intense during CD acute phase compared to the chronic phase and also more intense in the heart compared to the intestine. Considering animals infected with Colombian strain, leukocyte infiltration in the heart was classified as moderate in most animals during the acute phase, and as mild during the chronic phase of infection (*p* = 0.02). In the intestine, the degree of inflammation was classified as low both at the initial and late stages of infection (*p* > 0.05). Animals infected with the CL Brener strain presented persistent mild leukocyte infiltration in the heart and intestine during the acute and chronic phases (*p* > 0.05). A mild degree of inflammatory infiltration was observed in the cardiac and intestine tissues of animals infected with Y strain during CD acute phase, but the observed inflammation decreased to negative control levels during CD chronic phase (acute *versus* chronic phase – cardiac tissue *p* < 0.001; intestine tissue *p* = 0.02).

### Correlation and Principal Component Analysis

**Figure 6:**
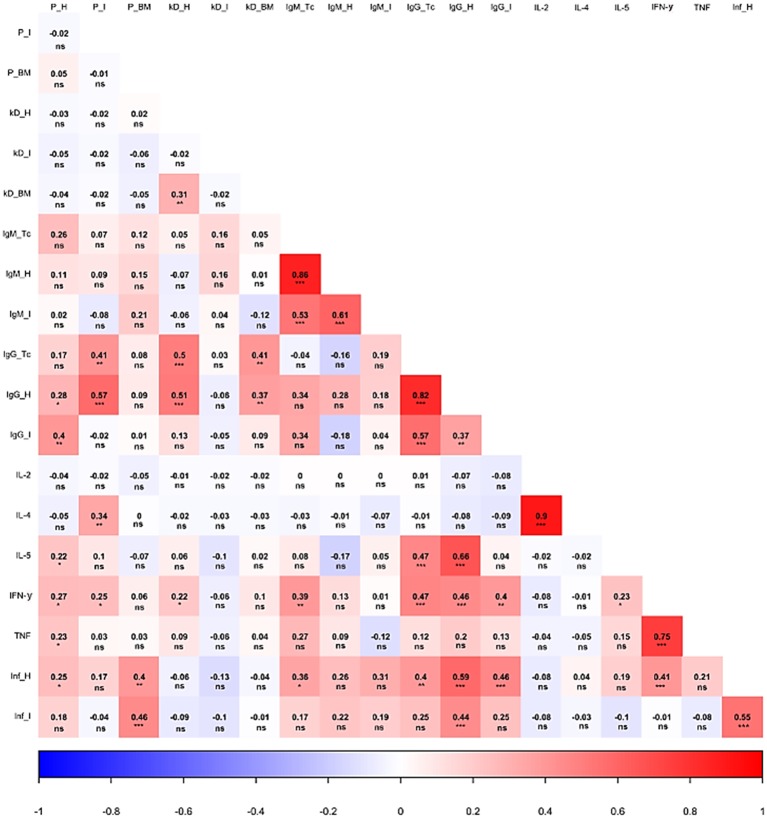
Correlation analysis of different parasite and host-related aspects during Chagas disease. The heat map shows the correlation of parasite load, kDNA integration, antibody titers, cytokine production, and inflammatory process during the infection. P: parasite load. kD: kDNA integration. Inf: inflammatory process. H: heart. I: intestine. BM: bone marrow. Tc: *Trypanosoma cruzi*. IL: interleukin. IFN: interferon γ. TNF: tumor necrosis factor. *p < 0.05, **p < 0.01, ***p < 0.001, ns – non significant.

The interaction among the parasite load in distinct anatomical sites, kDNA integration, antibody production against parasite and host antigens, cytokine production, as well as inflammatory infiltration was statistically analyzed and generated interesting insights. As shown in Figure 6, an important correlation between the parasite load and the activation of the cellular and humoral adaptive immune responses could be detected. For instance, a higher parasite load detected in the heart was significantly associated with a higher production of IgG directed to host cardiac (*p* < 0.05) and intestine (*p* < 0.01) proteins, but not to higher IgG raised against parasite proteins. A greater number of parasites in the cardiac tissue were also significantly connected to higher levels of IL-5, IFN-γ, and TNF (*p* < 0.05). In contrast, the intestine parasite burden was associated with an increase in IgG raised against *T. cruzi* (*p* < 0.01) and host cardiac proteins (*p* < 0.001). Interestingly, the intestine parasite load did not significantly alter IgG raised against host intestinal proteins. Still, regarding intestine parasite load, more frequent parasites in this tissue significantly increased the production of IL-4 (*p* < 0.01) and IFN-γ (*p* < 0.05). It was also observed that higher heart and bone marrow parasitism were associated with more intense inflammatory process observed in the heart (*p* < 0.05) and in the intestine (*p* < 0.001). Surprisingly, intestine parasitism failed to significantly correlate with the inflammatory process of this organ.

Parasite kDNA integration into host genome also influenced disease course, especially considering the host immune response. First, an association between heart and bone marrow integration rates was observed (*p* < 0.01). Higher kDNA integration at both sites was significantly associated with an altered humoral response, correlating to higher levels of IgG antibodies reactive to *T. cruzi* and host cardiac proteins (kDNA integration in the heart, *p* < 0.001; kDNA integration in bone marrow, *p* < 0.01). Finally, kDNA integration in the heart was also associated with higher IFN-γ levels (*p* < 0.05).

Humoral immune responses also significantly correlated with each other. Interestingly, the production of IgM or IgG reactive to a given antigen significantly correlated with superior production of these immunoglobulins against other antigens (e.g., higher IgM reactive against parasite proteins was associated to higher autoreactive IgM production). Also of note, the production of antibodies against *T. cruzi*, both in the acute or chronic phase, significantly correlated with IFN-γ production. Adding complexity into the scenario, the latter was associated with higher production of anti-host intestinal proteins IgG (*p* < 0.01). In addition, IL-5 showed a significant interaction with the production of anti-*T. cruzi* IgG, as well as anti-host cardiac proteins IgG (*p* < 0.001). A significant and directly proportional correlation was detected between different cytokines: IL-2 × IL-4 (*p* < 0.001), IL-5 × IFN-γ (*p* < 0.05), and IFN-γ × TNF (*p* < 0.001).

Antibody levels also influenced inflammatory infiltration in the tissue. A higher degree of lymphocytic infiltration in the heart was associated with the production of anti-*T. cruzi* IgM (*p* < 0.05), as well as anti-*T. cruzi* IgG (*p* < 0.01). Animals with a higher degree of leukocytes invading the heart also tended to present higher anti-host IgG (reactive to heart and intestine proteins; *p* < 0.001) and higher IFN-γ production (*p* < 0.001). Last, the inflammatory process of the intestine was directly associated with the inflammatory process of the heart (*p* < 0.001), as well as to the production of IgG reactive to host cardiac proteins.

**Figure 7:**
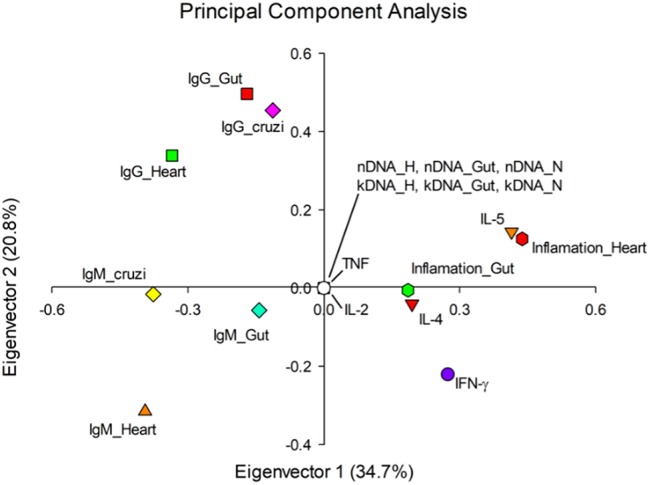
Eigenvectors of evaluated parameters of Chagas disease pathogenesis. The value in parentheses represents the variation that was explained by each eigenvector. Elements of the same quadrant behave similarly. nDNA: parasite load. kDNA: kDNA integration. Inf: inflammation. H: heart. I: intestine. BM: bone marrow. Tc: *Trypanosoma* cruzi. IL: interleukin. IFN: interferon γ. TNF: tumor necrosis factor.

The principal component analysis showed other important aspects of variable behavior throughout disease progression, the first two auto vectors explaining 55.5% of the variation observed (Figure 7). It was possible to note that the immunoglobulins act in a collaborative way in the host humoral response, both during the acute (IgM) and the chronic phase (IgG). In this context, IgM seemed to oppose the inflammation levels in different anatomical sites, mainly the cardiac tissue. Furthermore, the production of IFN-γ, IL-4, and IL-5 presented similar behavior to the inflammatory infiltration according to our analysis, possibly contributing to the onset of the inflammatory process.

## Discussion

In the Western Hemisphere, CD is responsible for the highest disease burden among parasitic diseases. Unfortunately, its physiopathology is still largely unknown, and treatment is still complex. In order to explain the lesions produced by *T. cruzi* infection, different theories have been created, accounting tissue lesion to parasite persistence or autoimmunity events. The difficulty in separating cause from effect in the context of CD clinical manifestations stems, in part, from contradictory information regarding the disease pathophysiology. For instance, the fact that only a percentage of the infected individuals develop clinical manifestations and the scarcity of amastigote nests in physical proximity to destroyed muscle fibers are still not completely understood (Teixeira et al., 1974; Root-Bernstein and Fairweather, 2015).

In the last 20 years, a range of review articles have discussed the evidence supporting or refuting the theories proposed to explain CD pathogenesis (Kierszenbaum, 1999, 2003, 2005; Monteón-Padilla et al., 2001; Engman and Leon, 2002; Bilate et al., 2003; Gironès et al., 2005, 2007; Cunha-Neto et al., 2006, 2009; Hyland and Engman, 2006; Marin-Neto et al., 2007; Bilate and Cunha-Neto, 2008; Bonney and Engman, 2008, 2015; Scharfstein et al., 2009; De Bona et al., 2018; Bonney et al., 2019). However, in most cases, the different elements that may contribute to the emergence of clinical manifestations of CD were analyzed separately. This kind of approach was not effective in establishing the mechanisms that trigger CD clinical manifestations, reinforcing the need for multiparametric analyses to better address the CD pathogenesis. In this respect, Santi-Rocca et al. (2017) have investigated CD outcome integrating parasite genetic background and some immune parameters, disclosing common patterns in CD pathogenesis. However, they did not look into the various aspects associated with CD autoimmunity. In our study, we infected male and female mice with Colombian, CL Brener and Y strains of *T. cruzi*, in order to perform a comprehensive analysis of how different elements of CD pathogenesis (parasite persistence, kDNA integration, molecular mimicry, and cytokines production) correlated with each other over time. Of note, even though it is difficult to correlate some aspects of CD pathogenesis in mice and humans, the murine model is widely used, enabling the acquisition of fundamental knowledge about several aspects of *T. cruzi* infection, such as CD outcome and pathophysiological processes, such as the production of autoantibodies and myocarditis (da Costa, 1999; Błyszczuk, 2019). Still, no mouse lineage can be considered as the unique classical model of the disease, since the host genetic background may influence the course of the disease (Pereira et al., 2014; Błyszczuk, 2019). Moreover, the use of mice infected by the intraperitoneal route can result in a different parasite distribution compared to more common and realistic routes of infection (Lewis et al., 2014; Silva-dos-Santos et al., 2017; Silberstein et al., 2018). Therefore, further multiparametric studies in humans are necessary to confirm our observations and achieve a better understanding of the CD pathophysiology.

### Parasite Load

First, we analyzed the presence of the parasites in cardiac, intestinal, and bone marrow tissues. As expected, direct observation of amastigote nests was only possible in cardiac tissue of mice infected with the Colombian strain, mostly during acute CD, in accordance with qPCR detection of the parasite DNA (nDNA) in cardiac tissue. Interestingly, such higher cardiac parasite burden was later associated with a greater inflammatory process. Both observations corroborate the notion that *T. cruzi* Colombian strain has histotropism to the myocardial and skeletal muscles, causing intense inflammatory infiltration, as well as altered cardiomyocyte function during the acute phase (Andrade, 1990; Camandaroba et al., 2006; Cruz et al., 2016).

Except for the CL Brener strain, the intestinal parasite load of the different experimental groups was similar to that found in the heart, according to qPCR data. Therefore, we hypothesize that the absence of amastigote nests found in the intestine may be a consequence to the low sensitivity of direct tissue observation. Considering the analysis performed with bone marrow samples, no significant difference was observed in the parasite load detected in the acute phase in the different experimental groups. This differs from what Melo and Brener (1978) reported, since they observed a high parasitism of the bone marrow from mice infected with Y strain, whereas with the CL Brener strain, few parasites resided in this tissue.

Our data demonstrated that there was no statistical difference between male and female mice parasite load, regardless of the infective strain or the infection phase. In a study evaluating the correlation of acute parasitemia by Y strain with longevity, it was found that males were more susceptible to death, although parasitemia was similar in both genders (Sanches et al., 2014). Thus, anatomical and hormonal distinctions between males and females do not seem to influence the establishment of the infection, although they may alter its outcome (Barretto et al., 1993).

The quantification of *T. cruzi* nDNA showed discrepant concentrations of parasites in some mice infected with Colombian and Y strains. A similar result was also obtained by Rodrigues-dos-Santos et al. (2018), who observed parasitic loads varying from 0.12 to 153.66 parasite equivalent/ml. As in our study, the authors detected higher parasitic loads in patients infected with TcII compared to patients infected with TcVI.

Correlation analysis revealed that higher parasitic loads in the heart and gut are associated with the production of autoantibodies against cardiac proteins, representing a direct link between parasite persistence and autoimmunity, possibly through molecular mimicry. On the other hand, the parasitemia in the bone marrow is linked to the inflammatory process of heart and intestine and not with autoreactive antibodies, indicating that possible changes of the myeloid cells, due to the presence of the parasite, may be altering the immune response regulation (Acosta Rodriguez et al., 2007; Müller et al., 2018).

### kDNA Integration

As aforementioned, the integration of parasite kDNA in the host genome is controversial in the literature, despite the fact that experimental evidence against such hypothesis has never been published so far. In the present work, we took several steps in order to validate the protocols used to detect kDNA integration during *in vivo* infection before assessing whether such event might influence other aspects of CD.

First, we validated an *in vitro* quantitative PCR (qPCR) protocol to detect the ratio of kDNA/nDNA in parasite samples of all strains investigated in the study. After validating primer efficiency and reproducibility of our analysis, such ratios were considered as thresholds for further experiments, kDNA/nDNA ratios greater than the established threshold being considered as integration events. Then, the kDNA/nDNA detection was validated *in vitro* using *T. cruzi-*infected macrophages, and accompanying kDNA/nDNA ratio at different time points, and also after benznidazole treatment, which effectively eliminated parasite nDNA, but not kDNA, supporting, once again, the occurrence of integration events in the context of *T. cruzi* infection. We then confirmed our observations performing genomic Southern blotting. Only after those steps were performed, we proceeded with the *in vivo* analysis of kDNA integration.

Our results showed that a higher percentage of animals had kDNA integration in the acute phase than in the chronic phase of the infection, especially in cardiac tissue. So, it is possible that the transfer of *T. cruzi* kDNA to the target cell genome may be an adaptive mechanism to invasion and survival in host cells. Actually, there are several examples of lateral transfer of DNA that benefit the invader. For instance, RNA viruses insert part of their genetic material into the host cell genome (Weiss, 2017). Another example is the extensive transfer of Wolbachia DNA into the genome of its host nematode *Brugia malayi*, since some regions of this DNA are biologically relevant to the bacterium (Ioannidis et al., 2013).

Curiously, when considering each tissue independently, we noticed that the total percentage of animals showing kDNA integration at 100 dpi decreased to a rate similar to the percentage of *T. cruzi*-infected individuals that develop the symptomatic chronic CD (30–40%) (Teixeira et al., 2011a). This data may be an indicator that the integration in one or more tissues can favor the emergence of clinical manifestations but requires further investigation.

Quantitative analysis of kDNA integrations showed that the number of integrations in cardiac tissue is related to the number of bone marrow integrations and that both correlate with the production of anti-*T. cruzi* and anti-cardiac IgG. This observation corroborates previous studies of kDNA integration in chickens, which developed cardiomyopathy even in the absence of the parasite, underscoring possible autoimmunity events (Teixeira et al., 2011b; Guimaro et al., 2014). The integration of kDNA into the bone marrow is another factor that may have an important role in the disease. Indeed, bone marrow transplantation in chickens that had kDNA integrated into their genomes inhibited the autoimmune response to the heart (Guimaro et al., 2014). The present data support the notion that such integration events occur and are active players in the context of CD physiopathology. Otherwise, kDNA integration would have failed to correlate to any other disease aspect significantly.

The use of multiparametric analysis in order to understand the possible role of kDNA integration into host genome importantly contributes to the field. Nevertheless, future studies investigating kDNA integration using modern molecular techniques like CRISPR/Cas-9 (Jiang and Doudna, 2017) may help elucidate this still controversial aspect of the disease in chickens, as well as other relevant species like mouse and humans.

### Immune Response Aspects

The investigation of immune response elements such as systemic cytokine levels greatly contributes toward disease comprehension, since cytokines are the main signaling molecules in this scenario. During early infection, high parasitemia resulted in activation of pro inflammatory response with high levels of TNF and IFN-γ being detected and corroborating the host attempt to eliminate the high parasite burden (Basso, 2013; Acevedo et al., 2018). The marked reduction in the systemic pro inflammatory cytokine dosage during the chronic phase of infection suggests that the exacerbated production of cytokines would not be the booster of CD pathogenesis, as proposed by the bystander activation theory (Gironès et al., 2005). Of interest, in infections where the complete elimination of the pathogen does not occur, the persistence of the antigen stimulates the emergence of “exhausted” T cells with reduced capacity to produce cytokines (Pack et al., 2018).

Importantly, we found that IFN-γ is associated with the production of autoreactive IgG against the heart and the inflammatory process of cardiac tissue. Thus, revealing a double-edged sword aspect for this cytokine. On one side, IFN-γ is largely recognized as an important mediator of host response against *T. cruzi* (Huang et al., 1993). On the other side, IFN-γ also seems to foster harmful autoimmunity. In accordance with our conclusion, Reifenberg et al. (2007) have also shown the importance of IFN-γ in autoimmune events. As described by Ferreira et al. (2014), IFN-γ favors the formation of inflammatory infiltrates in the cardiac tissue, but such effect goes hand in hand with detrimental effects toward cardiomyocytes.

Still considering the humoral immune response, we observed that, regardless of the sex, the immunoglobulins are initially produced against the parasite and, as the infection chronifies, autoantibodies are produced against heart and intestine (except for male mice infected with Y strain, which already produced autoantibodies at 30 dpi). In mice showing self-reaction to heart or intestine antigens during the acute phase, it is possible that autoimmunity is playing a role in tissue healing, rather than engendering more damage (Root-Bernstein and Fairweather, 2015). In this respect, it is known that natural IgM are spontaneously generated under physiological conditions. In the context of tissue lesion, such autoantibodies can accumulate in damaged intestinal and cardiac tissues, promoting tissue clearance. In this sense, autoreactivity may be considered both as being associated to tissue lesion, but also as a necessary step to pave the way for tissue repair (Nahrendorf et al., 2007; Ramos et al., 2012; Nunes-Silva et al., 2017; Sattler and Kennedy-Lydon, 2017). Since no correlation was detected between IgM-H or IgM-I, IgG-I and tissue inflammation, our data support the latter conception. Nevertheless, such hypothesis may be further investigated in future studies.

Besides, according to our data, the pathogenesis of cardiomegaly based on cross-reactive molecular mimicry (Bonney and Engman, 2015; De Bona et al., 2018) has a heterogeneous role when analyzing different *T. cruzi* strains, being more evident for Colombian and CL Brener strains. As hypothesized before, autoreactive T and B cells may be able to mediate homeostasis and healing and may not necessarily be harmful (Root-Bernstein and Fairweather, 2015). In this sense, the transformation of a benign autoimmunity into an autoimmune disease has been attributed to the balance of different cytokines (Rose, 1998). In the present study, we also observed that IFN-γ and IL-5 significantly correlate with self-recognition.

### Perspectives–A New Proposal of an Integrative Theory of Chagas Disease Pathogenesis

**Figure 8:**
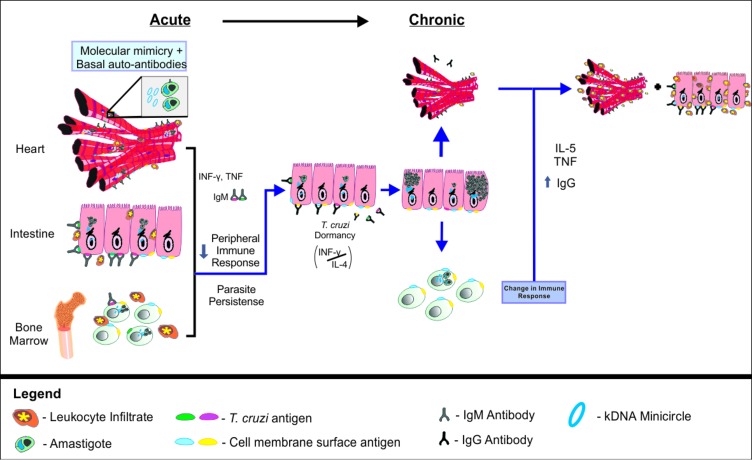
Integrative theory of Chagas disease pathogenesis. In early stages of acute Chagas disease, *T. cruzi* multiplies intensely and spreads through the body, activating the immune system and leading to the production of pro-inflammatory cytokines and IgM. As the *T. cruzi* invades host cells, kDNA minicircles are transferred to the host genome. Parasites in the bone marrow may compromise the production of mature B cells, favoring the persistence of the parasite in a latent state in the intestine. In this context, the balance of IL-4 and IFN-γ may be decisive for the maintenance or interruption of the *T. cruzi* latency during the asymptomatic chronic phase of Chagas disease. When parasite latency is disrupted, it may migrate to the bone marrow again and resume the pathological process, including the promotion of kDNA integration, autoantibody production, chronic tissue inflammation and lesion.

In the present study, we revisited CD pathology, performing classical observations of independent elements of CD pathogenesis (i.e., parasite load in relevant sites, cytokine production, and autoimmune recognition). Those observations mostly agree with previous studies and add very little to the field. Strikingly, though, when performing correlation and principal component analysis, we shed important light into how several elements of the disease interact over time and propose a new integrative theory for CD pathogenesis (Figure 8).

We propose that after initial infection, an intense multiplication of *T. cruzi* occurs, followed by parasite dissemination throughout the organism. The presence of the parasite is recognized by the host immune system, resulting in innate and adaptive immune response activation. Pro inflammatory cytokines, such as IFN-γ and TNF, are produced to eliminate the invading organism. Likewise, IgM is specifically raised against *T. cruzi* antigens, in an attempt of the host to eliminate infection. It is possible that this parasite-directed response is intrinsically associated to the production of autoreactive antibodies, which are initially relevant for tissue healing (Root-Bernstein and Fairweather, 2015). Gradually, as cells are increasingly invaded by *T. cruzi*, lateral transfer of kDNA minicircles from the parasite to the host genome occurs. We propose that this event represents an adaptive mechanism of the parasite to the host, exerting significant impact over the host immune response against the parasite, but also leveraging autoimmune reaction.

According to our holistic model, the presence of the parasite in the bone marrow results in alteration of immature B cells and reduction of mature B cells in peripheral sites (Acosta Rodriguez et al., 2007; Müller et al., 2018). This effect would, in turn, favor the persistence of the protozoan in the host. Still, even with less activation of B cells, the stress represented by the activation of the immune system may induce *T. cruzi* to shelter inside the host cells, entering into a latent state (Dumoulin and Burleigh, 2018). At least in mice, the preferential site where this event would occur is the intestine, as supported by the recent systems of real-time bioluminescence imaging (Lewis et al., 2014; Silberstein et al., 2018). Since IL-4 levels were significantly correlated to an increase of parasite load in the intestine, but not in the heart or in the bone marrow, it is possible to suggest that the production of IL-4 would support the parasite location in the gut, avoiding self-recognition. In this scenario, IFN-γ would have a dual role, promoting both host immunity response against *T. cruzi*, but also resulting in the production of harmful autoreactive antibodies. We believe that those events are kept in balance during the asymptomatic chronic phase of the disease.

After long periods spent in this equilibrated state, the breakdown of the IL-4/IFN-γ balance would then interrupt *T. cruzi* latency. During this process, which can occur intermittently throughout an individual’s life (Lewis and Kelly, 2016), the pathogen resumes multiplication and starts circulating through the organism, reaching the heart and bone marrow again, also resuming the pathological process. Considering the bone marrow, we suggest that myeloid cell damage potentiates the pathology in other organs by promoting inflammation (and not autoreactive antibodies). On the other hand, we propose that the integration of kDNA into bone marrow cells would promote autoreactive antibody production during the chronic phase of infection, contributing to the autoimmunity events observed in CD.

Parasite’s exit from the gastrointestinal tract would quickly reactivate the protective humoral immune response to infection, evidenced by higher serum anti-*T. cruzi* IgG. Once again, the process would be accompanied by tissue inflammation, as well as the production of autoantibodies. In this scenario, the altered bone marrow combined with molecular mimicry, and bystander activation, among others, would complement each other and result in autoimmunity. Importantly, the increased parasitaemia found in the heart would be connected to tissue lesion, engendered by direct parasite lesion, acute and chronic inflammation, and also by an autoimmune response, characterized by higher levels of anti-cardiac IgG.

## Data Availability

The raw data supporting the conclusions of this manuscript will be made available by the authors, without undue reservation, to any qualified researcher.

## Ethics Statement

This study was carried out in accordance with the guidelines for the use of laboratory animals established at our Institution. The protocol was approved by the University of Brasília Animal Research Ethics Committee (CEUA), under protocol 150406/2015.

## Author Contributions

MW, BD, and LH performed animals’ experiments. AM and AR performed culture experiments. MW, AM, TS, and TV performed qPCR analysis. AM performed genomic *Southern* blot tests. MW, AR, and NN performed serological tests. MW, BC, and TB performed cytokines dosage. MW, DR, and MH performed histopathology analysis. MW, AM, BD, and MH analyzed the data. JC and MH wrote the paper. All authors read and approved the final manuscript.

### Conflict of Interest Statement

The authors declare that the research was conducted in the absence of any commercial or financial relationships that could be construed as a potential conflict of interest.
