# Supplementary material for: Correlation of Parasite Burden, kDNA Integration, Autoreactive Antibodies, and Cytokine Pattern in the Pathophysiology of Chagas Disease
Source: Front Microbiol. 2019 Aug 21;10:1856. doi: 10.3389/fmicb.2019.01856 (PMC6712995; doi:10.3389/fmicb.2019.01856)
Supplement: Supplementary file 1 [file Data_Sheet_1.docx]

Supplementary Material

# Supplementary Figures and Tables

**Table S1. Experimental groups according to sex, infecting strain, and euthanasia phase.**

| **Group** | **Sex** | ***T. cruzi* strain** | **Euthanasia (dpi)** |
| --- | --- | --- | --- |
| **C-M** | Male | Colombian | 30 / 100 |
| **C-F** | Female | Colombian | 30 / 100 |
| **CL-M** | Male | CL Brener | 30 / 100 |
| **CL-F** | Female | CL Brener | 30 / 100 |
| **Y-M** | Male | Y | 30 / 100 |
| **Y-F** | Female | Y | 30 / 100 |
| **NC-M** | Male | Non infected | * |
| **NC-F** | Female | Non infected | * |

* Non infected animals were sacrificed at 90 days of age.


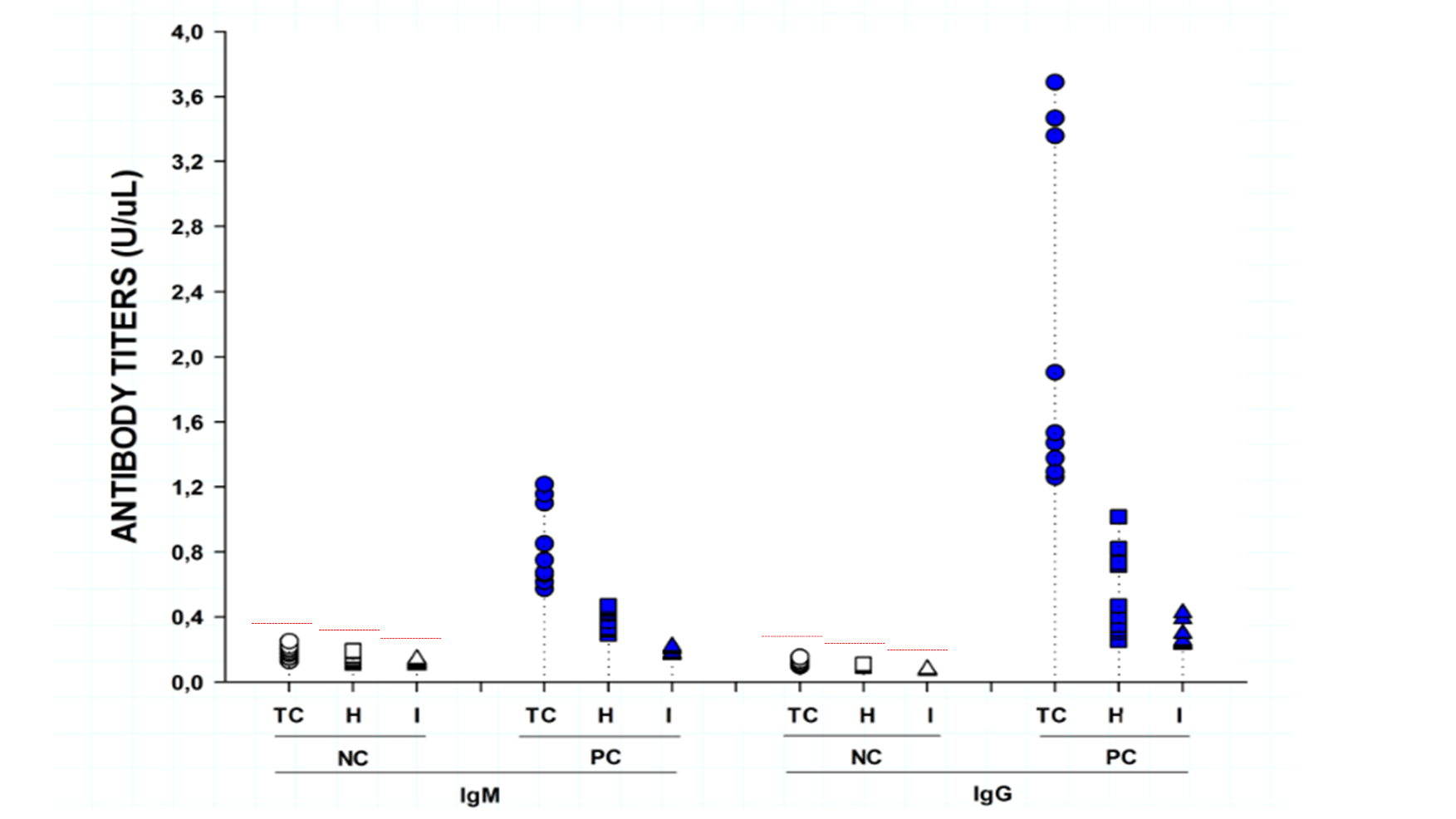


**Figure S1. Calculation of the ELISA’s cut-off based on negative control samples.** Mice were infected with 1x10^4^ intraperitoneal (i.p.) injection of trypomastigote forms of *T. cruzi.* At 30 and 100 dpi, serum samples were collected and immunoglobulin’s reactivity against parasite-derived (TC), heart-derived (H) and intestinal-derived (I) antigens was assessed by ELISA. IgM reactivity was assessed during the acute phase (30 dpi) and IgG reactivity was assessed during the chronic phase (100 dpi). Noninfected animals were used as negative controls (NC) to establish cut-off reactivity (Mean + 3 x Standard Deviation), indicated as red dashed lines. Absolute values are presented. PC: positive control (infected samples).


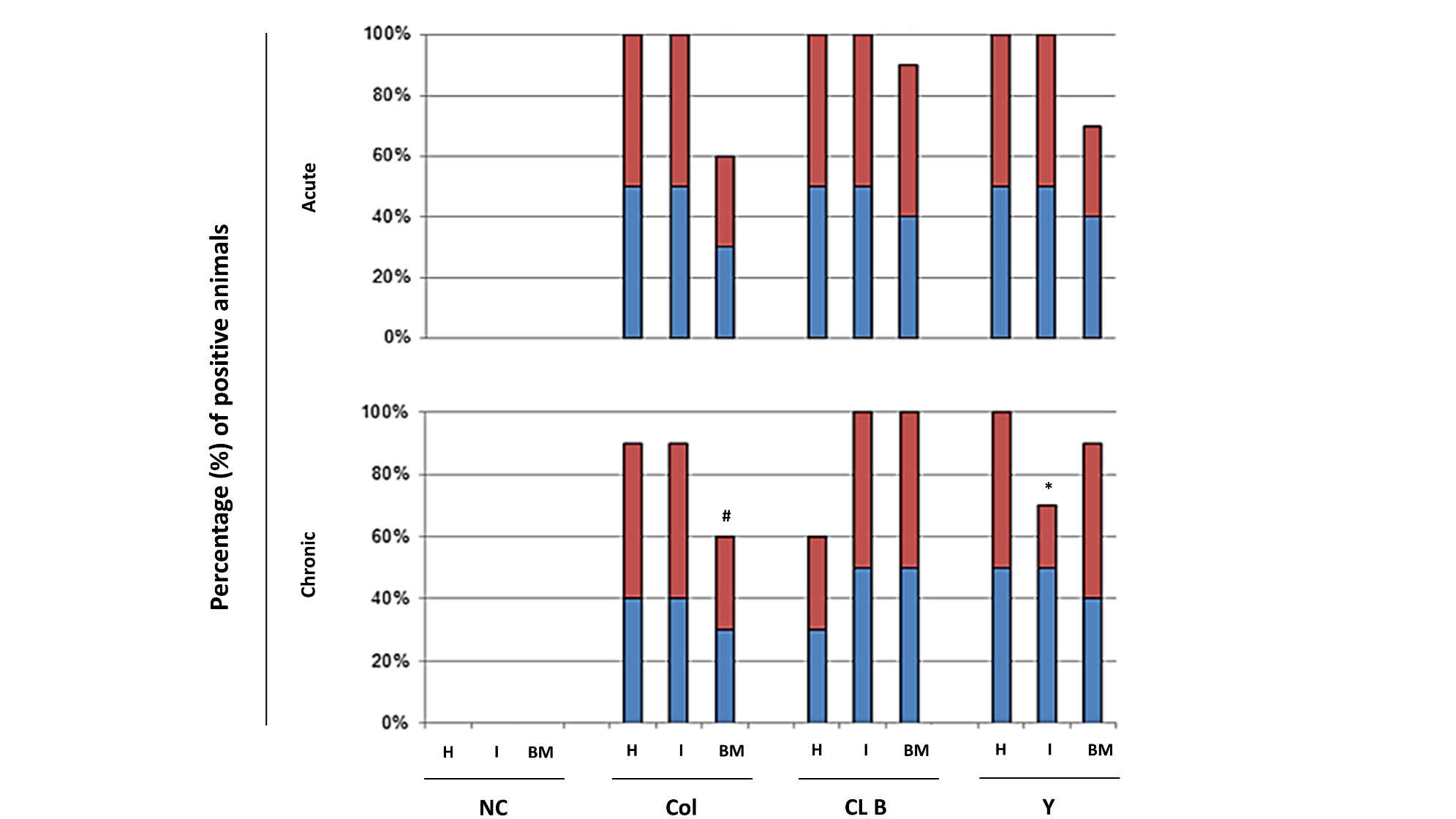


**Figure S2. Detection of *Trypanosoma cruzi* nuclear DNA during the acute and chronic phases of the infection**. After infection with different *T. cruzi* strains – Colombian (Col), CL Brener (CL B), or Y - , mice had their heart (H), intestine (I) and bone marrow (BM) tissues collected for DNA isolation and subsequent qPCR analysis. According to the chi-square test: Y-infected intestinal samples presented significantly decreased nDNA detection, compared to intestine samples from other groups, during the chronic phase (*p<0.05), and; Col-infected animals presented lower percentage of positive nDNA dectection in BM, compared to BM samples from other groups, also during the chronic phase (^#^p<0.05). Blue: male samples. Red: female samples. Statistically significant differences to the non-infected, negative control (NC) samples, were not presented.

**Table S2. Percentage of male *versus* female mice showing *Trypanosoma cruzi* nDNA amplification.**

|  | **Male** | **Female** | **p-value** |
| --- | --- | --- | --- |
| **Heart** | 67.50% a | 75.00% a | ns |
| **Intestine** | 72.50% a | 67.50% a | ns |
| **Bone marrow** | 55.00% a | 60.00% a | ns |

ns: non significant (p>0.05). According to the chi-square test, experimental groups with a statistically similar number of nDNA positive animals were designated with the same letter (a or b).

**
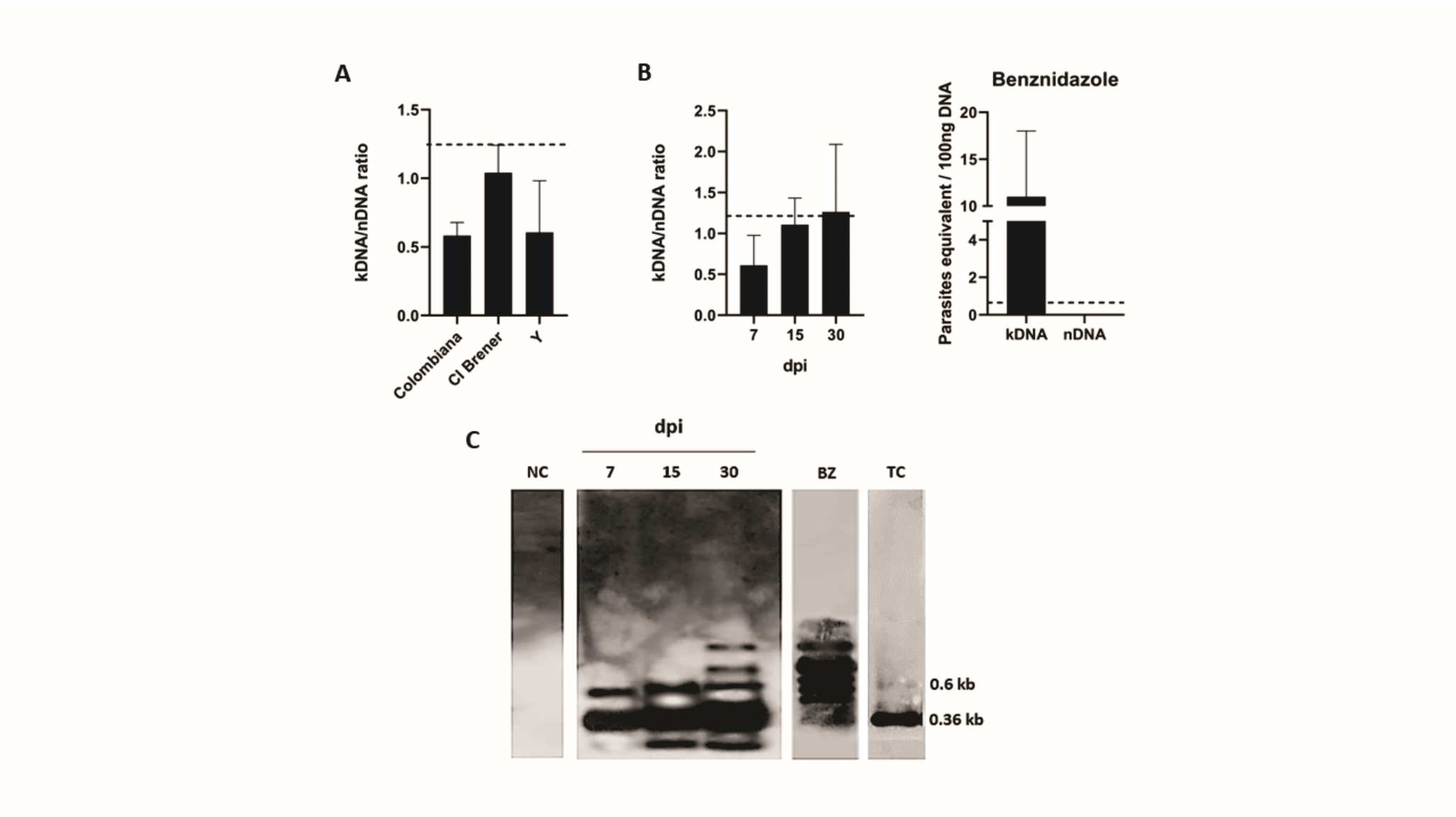
**

**Figure S3. Assessment of *Trypanosoma cruzi* kDNA integration. A**) Determination of the kDNA/nDNA ratio in *T. cruzi* amastigote pools by qPCR. The ratio of mitochondrial DNA (kDNA) and nuclear DNA (nDNA) of the Colombian, CL Brener and Y strains of *T. cruzi* were determined by amplification of the target sequences by qPCR. Values greater than the maximum ratio obtained for each strain represent the cut-off point from which integration of the kDNA minicircles was considered. **B)** J774A.1 cells infected with 10^6^  trypomastigotes had their DNA extracted at 7, 15, and 30 dpi to determine the kDNA/nDNA ratio by qPCR. Treatment with benznidazole eliminated parasitemia, resulting in no nuclear DNA amplification. In contrast, kDNA minicircle detection was maintained even after trypanocide treatments, suggesting integration into host genome. Dashed lines highlight kDNA/nDNA ratio threshold**. C)** *Southern* hybridization of NSiI digests with specific kDNA probe. Band profile of *T. cruzi* DNA is altered in infected macrophages, notedly at 30 dpi, suggesting integration events. The lack of the 0.36 kb band in beznidazole-treated cultures indicates that parasite has been eliminated, leaving kDNA minicircles sequences in the host genome (upper and lower bands). Data are presented as mean + standard deviation. NC, negative control. BZ, *T. cruzi* infected macrophages treated with benznidazole. TC, *T. cruzi*.

**
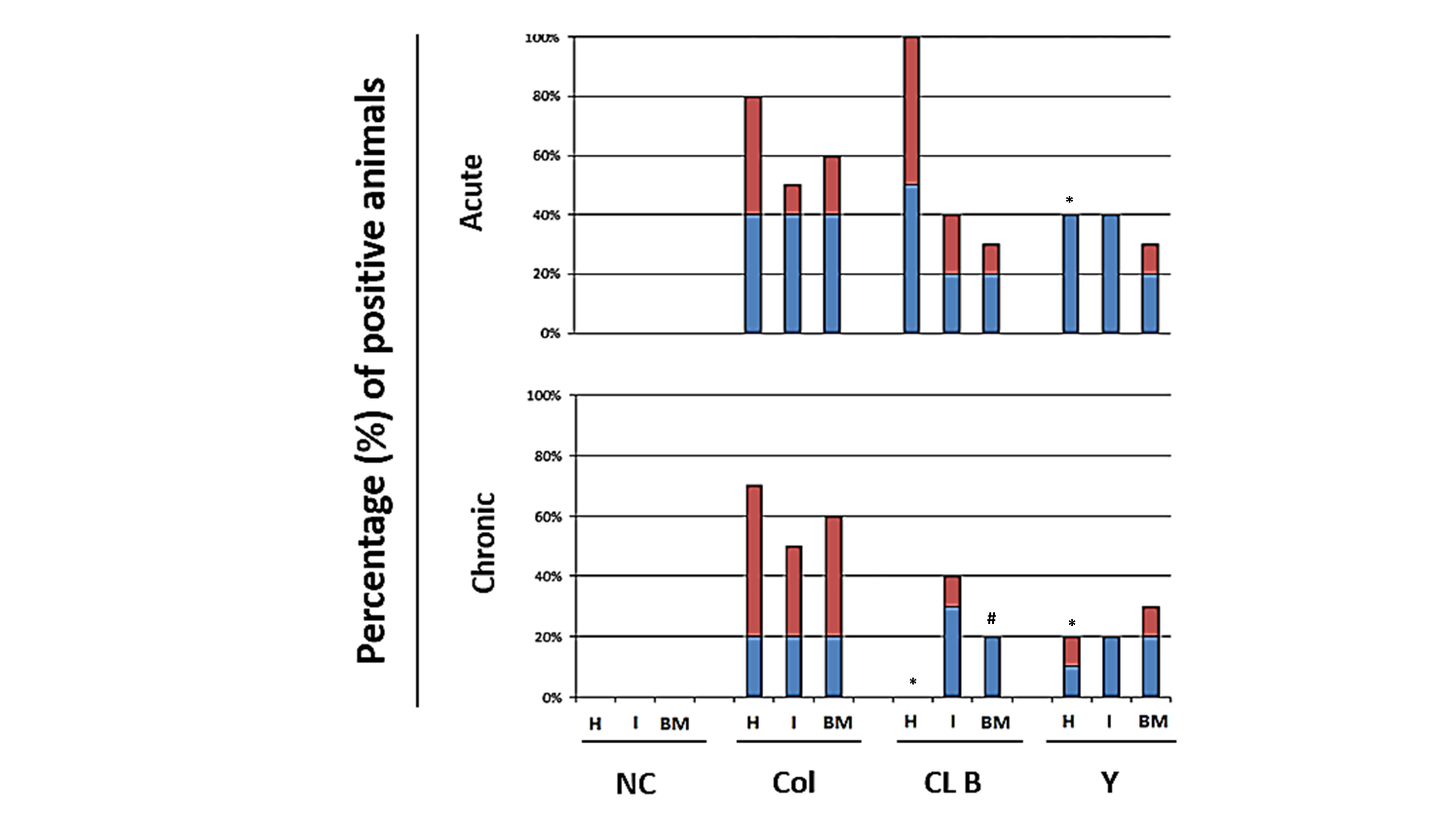
**

**Figure S4. Detection of *Trypanosoma cruzi* kDNA integration during the acute and chronic phases of the infection.** After infection with different *T. cruzi* strains – Colombian (Col), CL Brener (CL B), or Y -, the mice had their heart (H), intestine (I) and bone marrow (BM) tissues collected for DNA isolation and subsequent qPCR analysis. According to the chi-square test, heart samples from Y-infected mice presented significantly lower percentage of positive kDNA detection, compared to CL B heart samples, during the acute phase; also, during the chronic phase, both Y- and CL B-infected cardiac samples presented a significantly lower percentage of positive kDNA detection, compared to Col heart samples (*p<0.05). Bone marrow samples from CL B- and Y-infected mice presented significantly lower percentage of positive kDNA detection, compared to Col-infected group (^#^p<0.05). Blue: male samples. Red: female samples. Statistically significant differences to the non-infected, negative control (NC) samples were not presented.

|  | **Male** | **Female** | **p-value** |
| --- | --- | --- | --- |
| **Heart** | 53.33% a | 46.67% a | ns |
| **Intestine** | 56.67% a | 30.00% b | 0.04 |
| **Bone marrow** | 53.33% a | 46.67% a | ns |

**Table S3.** Percentage of male *versus* female mice showing *Trypanosoma cruzi* kDNA integration.

ns: non significant. According to the chi-square test, experimental groups with a statistically similar ratio of kDNA integration were designated with the same letter (a or b).

|  | **COLOMBIAN** | | | | **CL BRENER** | | | | **Y** | | | | **NC** | | | | |  | |
| --- | --- | --- | --- | --- | --- | --- | --- | --- | --- | --- | --- | --- | --- | --- | --- | --- | --- | --- | --- |
|  | Acute | | Chronic | | Acute | | Chronic | | Acute | | Chronic | | Acute | | Chronic | | | p-value | |
|  | M | SD | M | SD | M | SD | M | SD | M | SD | M | SD | M | SD | M | SD |  | |  |
| **IL-2** | 0.00 | 0.00 | 0.00 | 0.00 | 0.00 | 0.00 | 0.00 | 0.00 | 0.00 | 0.00 | 0.50 | 1.58 | 0.00 | 0.00 | 0.00 | 0.00 | ns | |  |
| **IL-4** | 0.09 | 0.27 | 0.00 | 0.00 | 0.16 | 0.50 | 0.00 | 0.00 | 0.00 | 0.00 | 0.40 | 1.25 | 0.03 | 0.10 | 0.03 | 0.10 | ns | |  |
| **IL-5** | 6.68 | 2.84 | 13.64 | 19.55 | 9.34 | 8.00 | 3.82 | 3.24 | 4.37 | 3.43 | 3.85 | 3.42 | 4.84 | 1.58 | 4.84 | 1.58 | ns | |  |
| **INF-γ** | 36.91a | 31.03 | 10.74b | 7.68 | 47.45a | 13.41 | 7.21b | 3.64 | 17.01b | 6.44 | 7.07b | 3.90 | 3.64b | 1.57 | 3.64b | 1.57 | <0.0001 | |  |
| **TNF** | 64.46 | 78.88 | 11.88 | 21.66 | 45.48 | 50.38 | 1.06 | 3.36 | 3.71 | 8.67 | 0.00 | 0.00 | 0.00 | 0.00 | 0.00 | 0.00 | Ns | |  |

**Table S4. Serum cytokine concentration (pg/mL) of *Trypanosoma cruzi* –infected animals during the chronic and acute phases of Chagas disease.**

NC: Negative control. M: mean. SD: standard deviation. ns: non significant. According to the ANOVA, experimental groups with a statistically similar concentration of an specific cytokine were designated with the same letter (a or b).


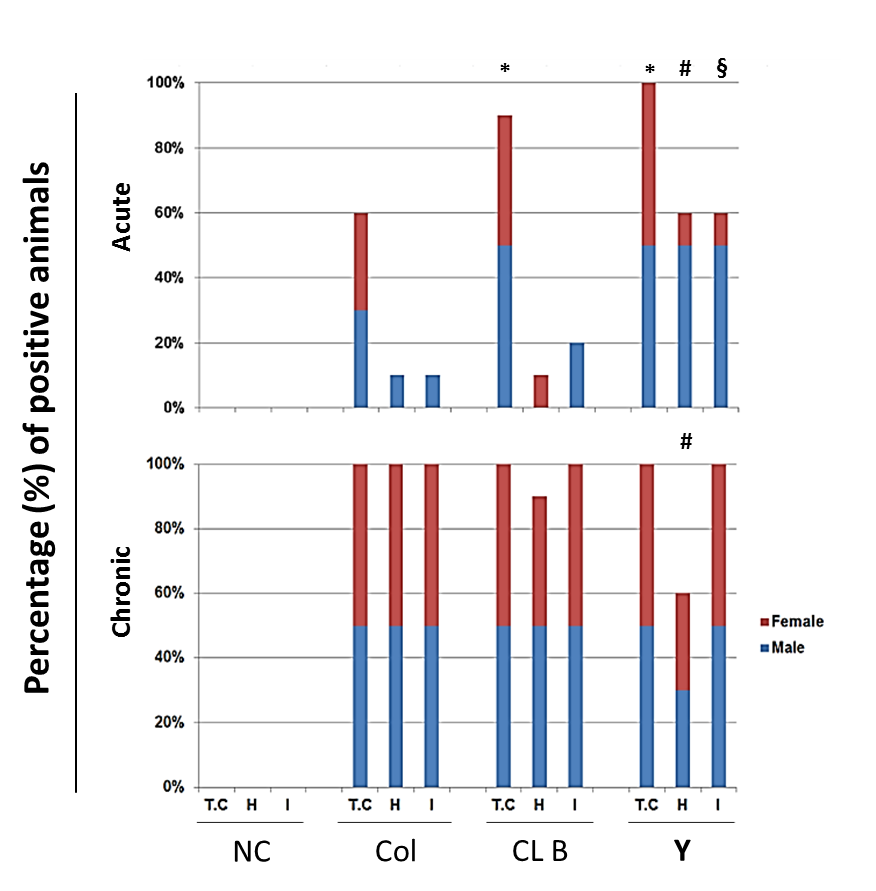


**Figure S5. Percentage of animals showing anti-*Trypanosoma cruzi* antibodies and autoantibodies**. Mice were infected with different *T. cruzi* (Tc) strains and evaluated according to serum IgM (30 dpi) and IgG (100 dpi) reactivity to *T. cruzi* antigens (T.C) and heart (H) and intestine (I) autoantigens. According to the chi-square test: CL B-infected and Y-infected animals presented significantly higher percentage of animals showing positive serum reaction to T.C antigens, compared to Col strain-infected mice during the acute phase (*p<0.05); Y-infected animals presented significantly higher percentage of animals showing positive serum reaction to H antigens during the acute phase and significantly lower during the chronic phase, compared to the other Tc strains (^#^p<0.05); Y-infected animals presented significantly higher percentage of animals showing positive serum reaction to H antigens during the acute phase, compared to animals infected with the other strains (^§^p<0.05). Statistically significant differences to the negative control (NC) samples were not presented.
